# Supplementary material for: Generation of Sesame Mutant Population by Mutagenesis and Identification of High Oleate Mutants by GC Analysis
Source: Plants (Basel). 2023 Mar 13;12(6):1294. doi: 10.3390/plants12061294 (PMC10055875; doi:10.3390/plants12061294)
Supplement: Supplementary file 1 [file plants-12-01294-s001.zip › Supplementary Figures.pdf]

```
(2)224663  0001  ATGGGAGCCGGAGGACGCATGTCTGATCCAACAACGAAAGACGAACAAAAGAAGAACCCCCTCCAACG
(4)278164  0001  ATGGGAGCCGGAGGACGCATGTCTGATCCAACAACGAAAGACGAACAAAAGAAGAACCCCCTCCAACG
(7)599435  0001  ATGGGAGCCGGAGGACGCATGTCTGATCCAACAACGAAAGACGAACAAAAGAAGAACCCCCTCCAACG
(8)599442  0001  ATGGGAGCCGGAGGACGCATGTCTGATCCAACAACGAAAGACGAACAAAAGAAGAACCCCCTCCAACG
(3)263470  0001  ATGGGAGCCGGAGGACGCATGTCTGATCCAACAACGAAAGACGAACAAAAGAAGAACCCCCTCCAACG
(1)188815  0001  ATGGGAGCCGGAGGACGCATGTCTGATCCGACAACGAAAGACGAACAAAAGAAGAACCCCCTCCAACG
(5)367899  0001  ATGGGAGCCGGAGGGCGCATGTCTGATCCAACAGCCAAAGACGAACAAAAGAAGAACCCCCTCCAACG
(6)490268  0001  ATGGGAGCTGGAGGACGCATGTCTGATCCAACAACCAAAGACGAACAAAAGAAGAACCCCCTCCATCG
                 --------*-----*--------------*---*-*-----------------------------*--

(2)224663  0069  GGTGCCTTACGCAAAGCCTCCATTCACACTCGGTGACATCAAGAAGGCCATTCCACCACACTGCTTCG
(4)278164  0069  GGTGCCTTACGCAAAGCCTCCATTCACACTCGGTGACATCAAGAAGGCCATTCCACCACACTGCTTCG
(7)599435  0069  GGTGCCTTACGCAAAGCCTCCATTCACACTCGGTGACATCAAGAAGGCCATTCCACCACACTGCTTCG
(8)599442  0069  GGTGCCTTACGCAAAGCCTCCATTCACACTCGGTGACATCAAGAAGGCCATTCCACCACACTGCTTCG
(3)263470  0069  GGTGCCTTACGCAAAGCCTCCATTCACACTCGGTGACATCAAGAAGGCCATTCCACCACACTGCTTCG
(1)188815  0069  GGTGCCTTACGCAAAGCCTCCGTTCACACTCGGTGACATCAAGAAGGCCATTCCACCACACTGCTTCG
(5)367899  0069  GGTGCCTTACGCAAAGCCTCCGTTCACACTTGGTGACATCAAGAAGGCCATTCCACCACACTGCTTTG
(6)490268  0069  GGTGCCCTACACAAAGCCTCCGTTCACACTAGGTGACATCAAGAAGGCCATTCCACCACACTGCTTTG
                 ------*---*----------*--------*-----------------------------------*-

(2)224663  0137  AGAGATCCGTCAGCCGTTCGTTCTCCTATGTCGTTTACGATCTCGTCATTGTTTTCCTTCTCTACTAC
(4)278164  0137  AGAGATCCGTCAGCCGTTCGTTCTCCTATGTCGTTTACGATCTCGTCATTGTTTTCCTTCTCTACTAC
(7)599435  0137  AGAGATCCGTCAGCCGTTCGTTCTCCTATGTCGTTTACGATCTCGTCATTGTTTTCCTTCTCTACTAC
(8)599442  0137  AGAGATCCGTCAGCCGTTCGTTCTCCTATGTCGTTTACGATCTCGTCATTGTTTTCCTTCTCTACTAC
(3)263470  0137  AGAGATCCGTCAGCCGTTCGTTCTCCTATGTCGTTTACGATCTCGTCATTGTTTTCCTTCTCTACTAC
(1)188815  0137  AGAGATCCGTCAGCCGTTCGTTCTCCTATGTCGTTTACGATCTCGTCATTGTTTTCCTTCTCTACTAC
(5)367899  0137  AGAGATCCGTCAGCCGTTCGTTCTCCTATGTCGTTTATGATCTCATCATCGTGTTCCTTCTCTACTAC
(6)490268  0137  AGAGATCTGTCAGCCGTTCGTTCTCCTATGTCGTTTACGATCTCGTCATCGTTTTCCTTCTCTACTAC
                 -------*-----------------------------*------*----*--*---------------

(2)224663  0205  ATTGCGACTTCTTACTTCCATCTGCTGCCATCCCCATACTGCTACCTAGCTTGGCCCATTTACTGGGC
(4)278164  0205  ATTGCGACTTCTTACTTCCATCTGCTGCCATCCCCATACTGCTACCTAGCTTGGCCCATTTACTGGGC
(7)599435  0205  ATTGCGACTTCTTACTTCCATCTGCTGCCATCCCCATACTGCTACCTAGCTTGGCCCATTTACTGGGC
(8)599442  0205  ATTGCGACTTCTTACTTCCATCTGCTGCCATCCCCATACTGCTACCTAGCTTGGCCCATTTACTGGGC
(3)263470  0205  ATTGCGACTTCTTACTTCCATCTGCTGCCATCCCCATACTGCTACCTAGCTTGGCCCATTTACTGGGC
(1)188815  0205  ATTGCGGCTTCTTACTTCCATCTGCTGCCATCCCCATACTGCTACCTAGCTTGGCCCATTTACTGGGC
(5)367899  0205  ATTGTGACTTCTTACTTCCATCTGCTGCCATCCCCGTACTGCTACCTAGCTTGGCCCATTTACTGGGC
(6)490268  0205  ATTGCGACTTCTTACATCCATCTGCTGCCATCCCCATACTCCTACCTAGCTTGGCCCATTTACTGGGC
                 ----*-*--------*-------------------*----*---------------------------

(2)224663  0273  TGTACAAGGCTGCGTTTGCACCGGAATCTGGGTCATTGCCCATGAATGTGGCCACCATGCATTCAGCG
(4)278164  0273  TGTACAAGGCTGCGTTTGCACCGGAATCTGGGTCATTGCCCATGAATGTGGCCACCATGCATTCAGCG
(7)599435  0273  TGTACAAGGCTGCGTTTGCACCGGAATCTGGGTCATTGCCCATGAATGTGGCCACCATGCATTCAGCG
(8)599442  0273  TGTACAAGGCTGCGTTTGCACCGGAATCTGGGTCATTGCCCATGAATGTGGCCACCATGCATTCAGCG
(3)263470  0273  TGTACAAGGCTGCGTTTGCACCGGAATCTGGGTCATTGCCCATGAATGTGGCCACCATGCATTCAGCG
(1)188815  0273  TGTACAAGGCTGTGTTTGCACCGGAATCTGGGTCATCGCCCATGAATGTGGCCACCATGCATTCAGCG
(5)367899  0273  TGTTCAAGGCTGTGTGTGCACCGGAATCTGGGTCATCGCCCATGAATGTGGCCACCATGCGTTCAGCG
(6)490268  0273  TTTTCAAGGCTGTGTTTGCACCGGAATCTGGGTCATCGCCCATGAATGTGGCCACCATGCATTCAGCG
                 -*-*--------*--*--------------------*-----------------------*-------

(2)224663  0341  ATTACCAGTGGCTTGACGACACAGTTGGCCTCATCCTGCACTCTGCCCTGCTCGTGCCCTATTTCTCA
(4)278164  0341  ATTACCAGTGGCTTGACGACACAGTTGGCCTCATCCTGCACTCTGCCCTGCTCGTGCCCTATTTCTCA
(7)599435  0341  ATTACCAGTGGCTTGACGACACAGTTGGCCTCATCCTGCACTCTGCCCTGCTCGTGCCCTATTTCTCA
(8)599442  0341  ATTACCAGTGGCTTGACGACACAGTTGGCCTCATCCTGCACTCTGCCCTGCTCGTGCCCTATTTCTCA
(3)263470  0341  ATTACCAGTGGCTTGACGACACAGTTGGCCTCATCCTGCACTCTGCCCTGCTCGTGCCCTATTTCTCA
(1)188815  0341  ATTACCAGTGGCTTGATGACACAGTTGGCCTCATCCTGCACTCTGCCCTGCTCGTGCCCTATTTCTCA
(5)367899  0341  ATTACCAGTGGCTTGATGACACAGTTGGCCTCATCCTGCACTCTGCCTTGCTTGTGCCCTATTTCTCT
(6)490268  0341  ATTACCAGTGGCTTGACGACACGGTTGGCCTCATCCTGCACTCTGCCCTGCTCGTGCCCTATTTCTCA
                 ----------------*-----*------------------------*----*--------------*
```

```
(2)224663  0409   TGGAAATACAGCCACCGCCGCCACCACTCCAACACTGGATCCCTTGAGCGTGACGAAGTCTTCGTCCC
(4)278164  0409   TGGAAATACAGCCACCGCCGCCACCACTCCAACACTGGATCCCTTGAGCGTGACGAAGTCTTCGTCCC
(7)599435  0409   TGGAAATACAGCCACCGCCGCCACCACTCCAACACTGGATCCCTTGAGCGTGACGAAGTCTTCGTCCC
(8)599442  0409   TGGAAATACAGCCACCGCCGCCACCACTCCAACACTGGATCCCTTGAGCGTGACGAAGTCTTCGTCCC
(3)263470  0409   TGGAAATACAGCCACCRCCGCCACCACTCCAACACTGGATCCCTTGAGCGTGACGAAGTCTTCGTCCC
(1)188815  0409   TGGAAATACAGCCACCGCCGCCACCACTCCAACACCGGATCCCTTGAGCGTGACGAAGTCTTCGTCCC
(5)367899  0409   TGGAAATACAGCCACCGCCGCCACCACTCCAACACCGGATCCCTTGAGCGTGATGAAGTCTTCGTCCC
(6)490268  0409   TGGAAATACAGCCACCGCCGTCACCACTCCAACACCGGATCCCTTGAGCGTGA1GAAGTCTT2GTCCC
                  ----------------+---*--------------*-----------------*--------+-----

(2)224663  0477   AAAGCCAAAATCCAGAGTCTCGTGGTACTCCAAATACTTGAACAATCCACTTGGCAGAGTCATCACAC
(4)278164  0477   AAAGCCAAAATCCAGAGTCTCGTGGTACTCCAAATACTTGAACAATCCACTTGGCAGAGTCATCACAC
(7)599435  0477   AAAGCCAAAATCCAGAGTCTCGTGGTACTCCAAATACTTGAACAATCCACTTGGCAGAGTCATCACAC
(8)599442  0477   AAAGCCAAAATCCAGAGTCTCGTGGTACTCCAAATACTTGAACAATCCACTTGGCAGAGTCATCACAC
(3)263470  0477   AAAGCCAAAATCCAGAGTCTCGTGGTACTCCAAATACTTGAACAATCCACTTGGCAGAGTCATCACAC
(1)188815  0477   AAAGCCGAAATCCAGAGTCTCGTGGTACTCCAAATACTTGAACAATCCACTTGGCAGAGTCATCACAC
(5)367899  0477   AAAGCCGAAATCCAGAGTCTCGTGGTACTCCAAATACTTGAACAATCCACTTGGCAGAGTCATCACAC
(6)490268  0477   AAAGCC3AAATCCAGAGTCTCGTGGTACTCCAAATACTTGAACAATCCACTTGGCAGAGTCAT4ACAC
                  ------*--------------------------------------------------------+----

(2)224663  0545   TTGTGGTTACTCTTACTCTCGGTTGGCCTCTATACTTGCTGTTTAATGTCTCTGGCAGGCCTTACAAC
(4)278164  0545   TTGTGGTTACTCTTACTCTCGGTTGGCCTCTATACTTGCTGTTTAATGTCTCTGGCAGGCCTTACAAC
(7)599435  0545   TTGTGGTTACTCTTACTCTCGGTTGGCCTCTATACTTGCTGTTTAATGTCTCTGGCAGGCCTTACAAC
(8)599442  0545   TTGTGGTTACTCTTACTCTCGGTTGGCCTCTATACTTGCTGTTTAATGTCTCTGGCAGGCCTTACAAC
(3)263470  0545   TTGTGGTTACTCTTACTCTCGGTTGGCCTCTATACTTGCTGTTTAATGTCTCTGGCAGGCCTTACAAC
(1)188815  0545   TTGTGGTTACTCTTACTCTCGGTTGGCCTCTATACTTGCTGTTTAATGTCTCTGGCAGGCCTTACAAC
(5)367899  0545   TTGTGGTTACTCTTACTCTTGGTTGGCCTCTATACTTGCTGTTTAATGTCTCTGGCAGGCCTTACAAC
(6)490268  0545   TTGTGGTTACTCTTACTCT5GGTTGGCCTCTATACTTGCTGTTTAATGTCTCTGGCAGGCCTTACAAC
                  -------------------*------------------------------------------------

(2)224663  0613   CGTTTTGCATGCCACTTTGACCCATATGGTCCAATATATAATGACCGTGAGAGACTTCAAATCTTCAT
(4)278164  0613   CGTTTTGCATGCCACTTTGACCCATATGGTCCAATATATAATGACCGTGAGAGACTTCAAATCTTCAT
(7)599435  0613   CGTTTTGCATGCCACTTTGACCCATATGGTCCAATATATAATGACCGTGAGAGACTTCAAATCTTCAT
(8)599442  0613   CGTTTTGCATGCCACTTTGACCCATATGGTCCAATATATAATGACCGTGAGAGACTTCAAATCTTCAT
(3)263470  0613   CGTTTTGCATGCCACTTTGACCCATATGGTCCAATATATAATGACCGTGAGAGACTTCAAATCTTCAT
(1)188815  0613   CGTTTTGCATGCCACTTTGACCCATATGGTCCAATATATAATGACCGTGAGAGACTTCAAATCTTCAT
(5)367899  0613   CGTTTTGCTTGCCACTTCGACCCGTACGGCCCAATATACAATGACCGTGAGAGACTTCAAATCTTCAT
(6)490268  0613   CGTTTTGCTTGCCACTTCGACCCGTACGGCCCAATATACAATGACCGTGAGAGACTTCAAATCTTCAT
                  --------*--------*-----*--*--*--------*-----------------------------

(2)224663  0681   CTCCGATGCTGGTATAATTGCTGCTGTATGTGTGCTTTATCGTGTTGCTTTGGTCAAAGGGTTGGCTT
(4)278164  0681   CTCCGATGCTGGTATAATTGCTGCTGTATGTGTGCTTTATCGTGTTGCTTTGGTCAAAGGGTTGGCTT
(7)599435  0681   CTCCGATGCTGGTATAATTGCTGCTGTATGTGTGCTTTATCGTGTTGCTTTGGTCAAAGGGTTGGCTT
(8)599442  0681   CTCCGATGCTGGTATAATTGCTGCTGTATGTGTGCTTTATCGTGTTGCTTTGGTCAAAGGGTTGGCTT
(3)263470  0681   CTCCGATGCTGGTATAATTGCTGCTGTATGTGTGCTTTATCGTGTTGCTTTGGTCAAAGGGTTGGCTT
(1)188815  0681   CTCCGATGCTGGTATAATTGCTGCTGTATGTGTGCTTTATCGTGTTGCTTTGGTCAAAGGGTTGGCTT
(5)367899  0681   CTCCGATGCTGGTATAATTGCTGCTGTATGTGTGCTTTATCGTGTTGCTTTGGTCAAAGGGTTGGCTT
(6)490268  0681   CTCCGATGCTGGTATAATTGCTGCTGTATGTGTGCTTTATCGTGTTGCTTTGGTCAAAGGGTTGGCTT
                  --------------------------------------------------------------------

(2)224663  0749   GGCTGGTATGTGTTTATGGGGTACCGTTACTCATTGTCAACGGTTTCCTTGTTTTGATCACATTCCTT
(4)278164  0749   GGCTGGTATGTGTTTATGGGGTACCGTTACTCATTGTCAACGGTTTCCTTGTTTTGATCACATTCCTT
(7)599435  0749   GGCTGGTATGTGTTTATGGGGTACCGTTACTCATTGTCAACGGTTTCCTTGTTTTGATCACATTCCTT
(8)599442  0749   GGCTGGTATGTGTTTATGGGGTACCGTTACTCATTGTCAACGGTTTCCTTGTTTTGATCACATTCCTT
(3)263470  0749   GGCTGGTATGTGTTTATGGGGTACCGTTACTCATTGTCAACGGTTTCCTTGTTTTGATCACATTCCTT
(1)188815  0749   GGCTGGTATGTGTTTATGGGGTACCGTTACTCATTGTCAACGGTTTCCTTGTTTTGATCACATTCCTT
(5)367899  0749   GGCTCGTATGTGTCTACGGGGTACCGTTACTCATTGTCAACGGGTTCCTTGTTTTGATCACATTCCTT
(6)490268  0749   GGCTCGTATGTGTCTACGGGGTACCGTTACTCATTGTCAACGGGTTCCTTGTTTTGATCACATTCCTT
                  ----*--------*--*--------------------------*------------------------
```

```
(2)224663  0817  CAGCACACTCACCCTTCGTTGCCGCACTATGATTCTTCCGAGTGGGACTGGCTAAGGGGAGCTCTTGC
(4)278164  0817  CAGCACACTCACCCTTCGTTGCCGCACTATGATTCTTCCGAGTGGGACTGGCTAAGGGGAGCTCTTGC
(7)599435  0817  CAGCACACTCACCCTTCGTTGCCGCACTATGATTCTTCCGAGTGGGACTGGCTAAGGGGAGCTCTTGC
(8)599442  0817  CAGCACACTCACCCTTCGTTGCCGCACTATGATTCTTCCGAGTGGGACTGGCTAAGGGGAGCTCTTGC
(3)263470  0817  CAGCACACTCACCCTTCGTTGCCGCACTATGATTCTTCCGAGTGGGACTGGCTAAGGGGAGCTCTTGC
(1)188815  0817  CAGCACACTCACCCTTCGTTGCCGCACTATGATTCTTCCGAGTGGGACTGGCTAAGGGGAGCTCTTGC
(5)367899  0817  CAGCACACTCACCCTTCATTGCCTCACTATGATTCTTCCGAGTGGGACTGGCTAAGGGGAGCTCTTGC
(6)490268  0817  CAGCACACTCACCCTTCATTGCCTCACTATGATTCTTCCGAGTGGGACTGGCTAAGGGGAGCTCTTGC
                 -----------------*-----*--------------------------------------------

(2)224663  0885  AACTGTCGACAGAGATTATGGGGTGCTAAATAAGGTGTTCCATAACATCACAGATACGCACGTGACTC
(4)278164  0885  AACTGTCGACAGAGATTATGGGGTGCTAAATAAGGTGTTCCATAACATCACAGATACGCACGTGACTC
(7)599435  0885  AACTGTCGACAGAGATTATGGGGTGCTAAATAAGGTGTTCCATAACATCACAGATACGCACGTGACTC
(8)599442  0885  AACTGTCGACAGAGATTATGGGGTGCTAAATAAGGTGTTCCATAACATCACAGATACGCACGTGACTC
(3)263470  0885  AACTGTCGACAGAGATTATGGGGTGCTAAATAAGGTGTTCCATAACATCACAGATACGCACGTGACTC
(1)188815  0885  AACTGTCGACAGAGATTATGGGGTGCTAAATAAGGTGTTCCATAACATCACAGATACGCACGTGACTC
(5)367899  0885  AACCGTCGACAGAGATTACGGGGTGCTAAATAAGGTGTTCCATAACATCACAGATACGCACGTGGCTC
(6)490268  0885  AACCGTCGACAGAGATTACGGGGTGCTAAATAAGGTGTTCCATAACATCACAGATACGCACGTGGCTC
                 ---*--------------*---------------------------------------------*---

(2)224663  0953  ACCACCTTTTCTCAACGATGCCACATTACCATGCAATGGAGGCAACTAAGGCAATCAAGCCCATACTG
(4)278164  0953  ACCACCTTTTCTCAACGATGCCACATTACCATGCAATGGAGGCAACTAAGGCAATCAAGCCCATACTG
(7)599435  0953  ACCACCTTTTCTCAACGATGCCACATTACCATGCAATGGAGGCAACTAAGGCAATCAAGCCCATACTG
(8)599442  0953  ACCACCTTTTCTCAACGATGCCACATTACCATGCAATGGAGGCAACTAAGGCAATCAAGCCCATACTG
(3)263470  0953  ACCACCTTTTCTCAACGATGCCACATTACCATGCAATGGAGGCAACTAAGGCAATCAAGCCCATACTG
(1)188815  0953  ACCACCTTTTCTCAACAATGCCACATTACCATGCAATGGAGGCAACTAAGGCAATCAAGCCCATACTG
(5)367899  0953  ACCACCTTTTCTCGACGATGCCACATTACCATGCAATGGAGGCAACTAAGGCAATCAAGCCAATACTG
(6)490268  0953  ACCACCTTTTCTCGACGATGCCACATTACCATGCAATGGAGGCAACTAAGGCAATCAAGCCAATACTG
                 -------------*--*--------------------------------------------*------

(2)224663  1021  GGCCAGTATTATCAGTTTGATGGAACCCCGTTTTACAAGGCGATGTGGAGGGAGGCAAAGGAATGTCT
(4)278164  1021  GGCCAGTATTATCAGTTTGATGGAACCCCGTTTTACAAGGCGATGTGGAGGGAGGCAAAGGAATGTCT
(7)599435  1021  GGCCAGTATTATCAGTTTGATGGAACCCCGTTTTACAAGGCGATGTGGAGGGAGGCAAAGGAATGTCT
(8)599442  1021  GGCCAGTATTATCAGTTTGATGGAACCCCGTTTTACAAGGCGATGTGGAGGGAGGCAAAGGAATGTCT
(3)263470  1021  GGCCAGTATTATCAGTTTGATGGAACCCCGTTTTACAAGGCGATGTGGAGGGAGGCAAAGGAATGTCT
(1)188815  1021  GGCCAGTATTATCAGTTTGATGGAACCCCGTTTTACAAGGCGATGTGGAGGGAGGCAAAGGAATGTCT
(5)367899  1021  GGCCAGTATTATCAGTTCGATGAAACCCCGTTTTACAAGGCGATGTGGAGGGAGGCAAAGGAATGTCT
(6)490268  1021  GGCCAGTATTATCAGTTCGATGAAACCCCGTTTTACAAGGCGATGTGGAGGGAGGCAAAGGAATGTCT
                 -----------------*-----*--------------------------------------------

(2)224663  1089  GTATGTCGAGCCAGACGAGAGTACTCCAGACAAGGGTGTATTCTGGTACAAGAACAAGTTCTGA
(4)278164  1089  GTATGTCGAGCCAGACGAGAGTACTCCAGACAAGGGTGTATTCTGGTACAAGAACAAGTTCTGA
(7)599435  1089  GTATGTCGAGCCAGACGAGAGTACTCCAGACAAGGGTGTATTCTGGTACAAGAACAAGTTCTGA
(8)599442  1089  GTATGTCGAGCCAGACGAGAGTACTCCAGACAAGGGTGTATTCTGGTACAAGAACAAGTTCTGA
(3)263470  1089  GTATGTCGAGCCAGACGAGAGTACTCCAGACAAGGGTGTATTCTGGTACAAGAACAAGTTCTGA
(1)188815  1089  GTATGTCGAGCCAGACGAGAGTACTCCAGACAAGGGTGTATTCTGGTACAAGAACAAGTTCTGA
(5)367899  1089  GTATGTCGAGCCAGACGAGAGTACTGCAGACAAGGGTGTGTTCTGGTACAAGAACAAGTTCTGA
(6)490268  1089  GTATGTCGAGCCAGACGAGAGTACTGCAGACAAGGGTGTGTTCTGGTACAAGAACAAGTTCTGA
                 -------------------------*-------------*------------------------
```

R = A/G heterozygote
1 = C or T
2 = C or T
3 = A or G
4 = G or C
5 = C or T

Figure S1A. Alignment of FAD2 gene DAN sequences from eight sesame accessions.

```
S. indicum R  092 VQGCVCTGIWVIAHECGHHAFSD YQWLDDTVGLILHSALLVPYFSW KYSHRRHHSNTGSLERDEVFVP KPKSRVSWYSKYLNNPLGRVITL
S. indicum 2  092 VQGCVCTGIWVIAHECGHHAFSD YQWLDDTVGLILHSALLVPYFSW KYSHRRHHSNTGSLERDEVFVP KPKSRVSWYSKYLNNPLGRVITL
S. indicum 4  092 VQGCVCTGIWVIAHECGHHAFSD YQWLDDTVGLILHSALLVPYFSW KYSHRRHHSNTGSLERDEVFVP KPKSRVSWYSKYLNNPLGRVITL
S. indicum 7  092 VQGCVCTGIWVIAHECGHHAFSD YQWLDDTVGLILHSALLVPYFSW KYSHRRHHSNTGSLERDEVFVP KPKSRVSWYSKYLNNPLGRVITL
S. indicum 8  092 VQGCVCTGIWVIAHECGHHAFSD YQWLDDTVGLILHSALLVPYFSW KYSHRRHHSNTGSLERDEVFVP KPKSRVSWYSKYLNNPLGRVITL
S. indicum 3  092 VQGCVCTGIWVIAHECGHHAFSD YQWLDDTVGLILHSALLVPYFSW KYSHHRHHSNTGSLERDEVFVP KPKSRVSWYSKYLNNPLGRVITL
S. indicum 1  092 VQGCVCTGIWVIAHECGHHAFSD YQWLDDTVGLILHSALLVPYFSW KYSHRRHHSNTGSLERDEVFVP KPKSRVSWYSKYLNNPLGRVITL
S. radiatum 5 092 VQGCVCTGIWVIAHECGHHAFSD YQWLDDTVGLILHSALLVPYFSW KYSHRRHHSNTGSLERDEVFVP KPKSRVSWYSKYLNNPLGRVITL
S. radiatum 6 092 FQGCVCTGIWVIAHECGHHAFSD YQWLDDTVGLILHSALLVPYFSW KYSHRRHHSNTGSLERDEVFVP KPKSRVSWYSKYLNNPLGRVITL
G. max 1      092 VQGCILTGVWVIAHECGHHAFSD YQLLDDIVGLVLHSGLLVPYFSW KYSHRRHHSNTGSLERDEVFVP KQKSCIKWYSKYLNNPPGRVLTL
G. max 2      092 VQGCILTGVWVIAHECGHHAFSD YQLLDDIVGLILHSALLVPYFSW KYSHRRHHSNTGSLERDEVFVP KQKSCIKWYSKYLNNPPGRVLTL
A. duranensis 092 IQGCILTGVWVIAHECGHHAFSK YQLVDDMVGLTLHSCLLVPYFSW KISHRRHHSNTGSLDRDEVFVP KPKSKVSWYNKYMNNPPGRAISL
A. hypogaea 1 092 IQGCILTGVWVIAHECGHHAFSK YQLVDDMVGLTLHSCLLVPYFSW KISHRRHHSNTGSLDRDEVFVP KPKSKVSWYNKYINNPPGRAISL
A. hypogaea 2 092 IQGCILTGVWVIAHECGHHAFSK YQLVDDMVGLTLHSCLLVPYFSW KISHRRHHSNTGSLDRNEVFVP KPKSKVSWYNKYMNNPPGRAISL
A. hypogaea 3 092 IQGCILTGVWVIAHECGHHAFSK YQLVDDMVGLTLHSCLLVPYFSW KISHRRHHSNTGSLDRDEVFVP KPKSKVSWYNKYMNNPPGRAISL
A. monticola  092 IQGCILTGVWVIAHECGHHAFSK YQLVDDMVGLTLHSCLLVPYFSW KISHRRHHSNTGSLDRDEVFVP KPKSKVSWYNKYMNNPPGRAISL
A. ipaensis   092 IQGCILTGVWVIAHECGHHAFSK YQLVDDMVGLTLHSCLLVPYFSW KISHRRHHSNTGSLDRDEVFVL KPKSKVSWYNKYMNNPPGRAISL
```

Figure S1B. Identification of an amino acid substitution of R142H in the H-Box2 by comparison of the partial deduced amino acid sequences from *FAD2* genes among species from three different genera.

The yellow color highlighted amino acids is the H-Box2 in the partial deduced amino acid sequences started from position 92. The red color H is shown the amino acid substitution of R142H. *S. indicum* is from sesame. *S. radiatum* is from sesame wild species. *G. max* is from soybean. *A. hypogaea* is from peanut. The rest is from peanut wild species.
