# Supplementary material for: Generation of Sesame Mutant Population by Mutagenesis and Identification of High Oleate Mutants by GC Analysis
Source: Plants (Basel). 2023 Mar 13;12(6):1294. doi: 10.3390/plants12061294 (PMC10055875; doi:10.3390/plants12061294)
Supplement: Supplementary file 1 [file plants-12-01294-s001.zip › Supplementary Tables.pdf]

Table S1. Comparison of fatty acid profiles among different *FAD2* genotypes

Plant number	PI number	Genotype (G425A)	C16:0 (%)	C16:1 (%)	C18:0 (%)	C18:1 (%)	C18:2 (%)	C18:3 (%)	C20:0 (%)	C20:1 (%)	C22:0 (%)	C24:0 (%)
**3-1**	263470	A/A	9.40	0.19	4.76	52.78	31.49	0.42	0.58	0.19	0.13	0.08
**3-3**	263470	A/A	9.19	0.16	4.84	52.77	31.66	0.35	0.61	0.20	0.16	0.08
**3-10**	263470	A/A	9.11	0.15	5.07	53.18	31.09	0.37	0.63	0.19	0.16	0.08
**3-11**	263470	A/A	8.04	0.12	4.42	56.59	29.44	0.37	0.58	0.23	0.15	0.08
**3-22**	263470	A/A	8.96	0.15	5.36	54.88	29.24	0.35	0.64	0.20	0.16	0.08
**3-23**	263470	A/A	8.83	0.15	5.13	55.17	29.32	0.36	0.64	0.20	0.16	0.08
**3-6**	263470	G/A	9.11	0.15	4.89	47.17	37.32	0.35	0.61	0.19	0.16	0.08
**2**	224663	G/G	8.82	0.13	4.88	39.22	45.69	0.33	0.55	0.19	0.13	0.08

PI: plant introduction; G425A: natural mutation position in the DAN coding sequence of *FAD2* gene.

Table S2. Planting time, early plant stature, flowering time, and harvest time in 2022 in greenhouse

Plant number	Planting time	Early plant stature[*]	Flowering time[¥]	Harvested time	Plant number	Planting time	Early plant stature[*]	Flowering time	Harvested time
**Check PI263470 (A/A)**					**M_7 965_2_36_5_3_36**				
1	04/28	Normal	05/20	07/18	1	04/28	Normal	05/20	07/24
2	04/28	Normal	05/20	07/18	2	04/28	Normal	05/20	07/24
3	04/28	Normal	05/20	07/18	3	04/28	Normal	05/20	07/24
4	04/28	Normal	05/20	07/18	4	04/28	Normal	05/20	07/24
5	04/28	Normal	05/20	07/18	5	04/28	Normal	05/20	07/24
6	04/28	Normal	05/20	07/18	6	04/28	Normal	05/20	07/24
7	04/28	Normal	05/20	07/18	7	04/28	Small	05/20	07/24
8	04/28	Normal	05/20	07/18	8	04/28	Normal	05/20	07/24
**M_7 965_2_33_7_6_10**					**M_7 965_2_11_6_2_29**				
1	04/28	Normal	06/28	08/18	1	04/28	Normal	05/21	07/26
2	04/28	Normal	06/28	08/18	2	04/28	Normal	05/21	07/26
3	04/28	Normal	06/28	08/18	3	04/28	Normal	05/21	07/26
4	04/28	Normal	06/28	08/18	4	04/28	Normal	05/21	07/26
5	04/28	Normal	06/28	08/18	5	04/28	Normal	05/21	07/26
6	04/28	Normal	06/28	08/18	6	04/28	Normal	05/21	07/26
7	04/28	Normal	06/28	08/18	7	04/28	Normal	05/21	07/26
8	04/28	Normal	06/28	08/18	8	04/28	Normal	05/21	07/26
**M_7 915_1_35_4_3_2**					**M_7 915_1_35_5_5_3**				
1	04/28	Normal	05/21	07/15	1	04/28	Normal	05/21	07/13
2	04/28	Normal	05/21	07/15	2	04/28	Normal	05/21	07/13
3	04/28	Normal	05/21	07/15	3	04/28	Normal	05/21	07/13
4	04/28	Normal	05/21	07/15	4	04/28	Normal	05/21	07/13
5	04/28	Normal	05/21	07/15	5	04/28	Normal	05/21	07/13
6	04/28	Normal	05/21	07/15	6	04/28	Normal	05/21	07/13
7	04/28	Normal	05/21	07/15	7	04/28	Normal	05/21	07/13
8	04/28	Normal	05/21	07/15	8	04/28	Normal	05/21	07/13
**M_8 200_1_11_15_3_5_21**									

1	04/28	Leafy	05/21	07/28	5	04/28	Leafy	05/21	07/28
2	04/28	Leafy	05/21	07/28	6	04/28	Leafy	05/21	07/28
3	04/28	Leafy	05/21	07/28	7	04/28	Leafy	05/21	07/28
4	04/28	Small	05/21	07/28	8	04/28	Leafy	05/21	07/28

*Early plant stature was observed after planting 35 days (on June 2, 2022).

¥Harvested time was recorded as the time for finished harvesting all capsules from the plant.

Table S3. Average value and range each fatty acid of sesame mutant lines

Genotype	PI 263470	M_8 200-1	M_7 915-2	M_7 915-3	M_7 965-29	M_7 965-10	M_7 965-36
**C16:0 (%)**	9.54 (9.34-9.68)	6.80 (6.36-7.17)	7.48 (7.08-7.80)	8.32 (8.02-8.73)	9.40 (9.00-9.75)	9.24 (8.99-9.47)	9.41 (8.96-9.62)
**C16:1 (%)**	0.17 (0.15-0.18)	0.17 (0.16-0.18)	0.19 (0.16-22)	0.18 (0.16-0.20)	0.17 (0.15-0.18)	0.18 (0.18-0.19)	0.17 (0.16-0.17)
**C17:0 (%)**	0.05 (0.05-0.06)	0.09 (0.08-0.11)	0.12 (0.11-0.14)	0.08 (0.06-0.09)	0.07 (0.06-0.08)	0.06 (0.05-0.07)	0.08 (0.07-0.10)
**C17:1 (%)**	0 (0)	0.06 (0.05-0.07)	0 (0)	0 (0)	0.06 (0.05-0.06)	0.05 (0.05-0.05)	0.06 (0.05-0.06)
**C18:0 (%)**	5.28 (5.09-5.44)	7.20 (6.42-7.76)	7.08 (6.61-7.53)	7.81 (7.31-8.25)	5.05 (4.73-5.42)	4.65 (4.24-5.06)	5.50 (5.15-6.04)
**C18:1 (%)**	56.88 (55.59-58.35)	72.10 (70.42-73.20)	75.41 (74.20-77.30)	72.89 (70.25-73.91)	69.70 (68.22-71.52)	69.20 (68.35-71.77)	71.21 (69.31-74.30)
**C18:2 (%)**	26.82 (25.40-28.17)	12.16 (10.51-14.76)	7.77 (6.85-8.58)	8.85 (7.37-11.43)	14.29 (12.49-15.60)	15.37 (12.71-17.52)	12.22 (8.83-14.32)
**C18:3 (%)**	0.25 (0.27-0.30)	0.28 (0.26-0.30)	0.46 (0.38-0.53)	0.48 (0.44-0.52)	0.27 (0.26-0.29)	0.29 (0.26-0.32)	0.29 (0.27-0.32)
**C20:0 (%)**	0.60 (0.59-0.61)	0.80 (0.69-0.87)	0.92 (0.85-0.99)	0.93 (0.89-0.96)	0.63 (0.59-0.66)	0.58 (0.53-0.62)	0.66 (0.61-0.73)
**C20:1 (%)**	0.21 (0.20-0.21)	0.28 (0.26-0.33)	0.26 (0.24-0.28)	0.23 (0.22-0.24)	0.21 (0.20-0.21)	0.21 (0.20-0.21)	0.22 (0.21-0.24)
**C22:0 (%)**	0.13 (0.12-0.13)	0.16 (0.14-0.17)	0.18 (0.16-0.20)	0.16 (0.15-0.17)	0.13 (0.13-0.15)	0.21 (0.11-0.13)	0.14 (0.13-0.16)
**C24:0 (%)**	0.07 (0.07-0.08)	0.08 (0.07-0.09)	0.15 (0.13-0.17)	0.13 (0.12-0.15)	0.08 (0.07-0.08)	0.07 (0.06-0.08)	0.07 (0.07-0.08)

For each fatty acid, the first value was the average value, and the range of average value was in the bracket in each cell. Except for the average values of M_8 200 from four plants, all the other average values were from eight plants.

M_8 200-1, M_8 200-1-11-15-3-5-21; M_7 915-2, M_7 915-1-35-4-3-2; M_7 915-3; M_7 915-1-35-5-5-3;

M_7 965-29; M_7 965-2-11-6-2-29; M_7 965-10; M_7 965-2-33-7-6-10; M_7 965-36; M_7 965-2-36-5-3-36.
